# Sociodemographic characteristics determine download and use of a Corona contact tracing app in Germany—Results of the COSMO surveys

**DOI:** 10.1371/journal.pone.0256660

**Published:** 2021-09-02

**Authors:** Eva Grill, Sarah Eitze, Freia De Bock, Nico Dragano, Lena Huebl, Patrick Schmich, Lothar H. Wieler, Cornelia Betsch

**Affiliations:** 1 Institute for Medical Information Processing, Biometry and Epidemiology, Ludwig-Maximilians University München, München, Germany; 2 Media and Communication Science, University of Erfurt, Erfurt, Germany; 3 Federal Centre for Health Education (Bundeszentrale für gesundheitliche Aufklärung (BZgA)), Cologne, Germany; 4 Institute of Medical Sociology, Medical Faculty and University Hospital Düsseldorf, Heinrich-Heine-University Düsseldorf, Düsseldorf, Germany; 5 University Medical Centre Hamburg-Eppendorf, I. Department of Medicine, Division Tropical Medicine, Hamburg, Germany; 6 Robert Koch-Institute, Berlin, Germany; University of Salamanca, SPAIN

## Abstract

During the SARS-CoV-2 pandemic mobile health applications indicating risks emerging from close contacts to infected persons have a large potential to interrupt transmission chains by automating contact tracing. Since its dispatch in Germany in June 2020 the Corona Warn App has been downloaded on 25.7 Mio smartphones by February 2021. To understand barriers to download and user fidelity in different sociodemographic groups we analysed data from five consecutive cross-sectional waves of the COVID-19 Snapshot Monitoring survey from June to August 2020. Questions on the Corona Warn App included information on download, use, functionality, usability, and consequences of the app. Of the 4,960 participants (mean age 45.9 years, standard deviation 16.0, 50.4% female), 36.5% had downloaded the Corona Warn App. Adjusted analysis found that those who had downloaded the app were less likely to be female (Adjusted Odds Ratio for men 1.16 95% Confidence Interval [1.02;1.33]), less likely to be younger (Adjusted Odds Ratio for age 18 to 39 0.47 [0.32;0.59] Adjusted Odds Ratio for age 40 to 64 0.57 [0.46;0.69]), less likely to have a lower household income (AOR 0.55 [0.43;0.69]), and more likely to live in one of the Western federal states including Berlin (AOR 2.31 [1.90;2.82]). Willingness to disclose a positive test result and trust in data protection compliance of the Corona Warn App was significantly higher in older adults. Willingness to disclose also increased with higher educational degrees and income. This study supports the hypothesis of a digital divide that separates users and non-users of the Corona Warn App along a well-known health gap of education, income, and region.

## Introduction

A primary goal of current containment strategies of severe acute respiratory syndrome coronavirus 2 (SARS-CoV-2) is the reduction of its burden of disease [1], and one particular goal is to keep the incidence of new infections at a level that facilitates epidemic control until vaccination or effective treatment will become available [2].Tracing, testing, informing and isolating cases’ contacts has been established in many countries as one of many effective measures to interrupt infection chains.

The infectious characteristics of SARS-CoV-2 indicate that a substantial percentage of transmissions occur through infected persons in the presymptomatic stage or through asymptomatic cases. Therefore, an effective containment strategy for COVID-19 must focus on rapidly informing contact persons of cases before they become infectious [3]. While it is advised that each confirmed case directly informs their personal contacts, this mandatory task is mainly performed by local Public Health authorities many of whom use manual tracing methods. This procedure is of utmost importance, but it is time-consuming and the quality of manual contact tracing largely depends on resources of the local Public Health institutions.

To add to the well-established and successful manual contact tracing strategies, additional digital tools would enable to test and trace contacts without delay. The idea is that manual contact tracing can be supplemented by appropriate and effective mHealth applications (apps). These apps might have the potential to interrupt transmission chains by tracing and identifying presymptomatic infections. Modelling studies have shown that this can be an essential component of contact tracing and infection control [4]. As of Dec 23, 2020, over 25 national tracing apps have been launched worldwide [5]. The German Ministry of Health dispatched a Corona tracing app in June 2020 (“Corona Warn App”, CWA) which indicates potential infection risks emerging from close contacts to infected persons. CWA is based on Bluetooth proximity tracing and an epidemiological risk algorithm. It advises on subsequent actions such as self-observation of symptoms, self-isolation or getting tested. CWA had a successful start with over 15 Mio downloads within the first four weeks, (18% of the German population, 26% of the 57.7 Mio German smart phone users), then stagnating, and being at 25.7 Mio downloads as of Feb 23, 2021 [6].

To be an acceptable and indispensable part of the German containment strategy, several preconditions for CWA have been discussed. Necessarily, one of the preconditions is efficacy and effectiveness, i.e., reach and timeliness of identifying potential contacts of cases, and cases avoided through timely action [7]. Effectiveness in real life on a national level, however, is further defined by additional parameters, namely fidelity and uptake. Fidelity of use refers to the basic actions proposed by the app: self-isolate and undergo testing if necessary, and report a positive test result immediately through the app. Ultimately, uptake is one of the most salient parameters. Based on modelling studies [8] a necessary uptake by 80% of smart-phone users of a population was posited if digital contact tracing would be the only counter-infection measure. In European countries where several additional measures such as social distancing and manual contact tracing are in place a contact tracing app will arguably be effective even if uptake is lower. Still, the app needs to be accessible independently of socioeconomic status, education, or age. In this context it has been noted repeatedly [9–11] that digital health applications including mobile phone health apps have the potential to deepen social inequalities in health. Persons with poor health, low income or education, and older adults are more likely to have low digital skills and less technical resources limiting their use of and access to effective eHealth interventions [12]. At the same time these groups are also more vulnerable to infection and severe COVID-19 disease [13, 14]. Also, persons with a history of discrimination tend to be more vulnerable and less likely to accept digital contact tracing [15, 16]. Thus, a socially differential use of tracing apps may further aggravate existing inequalities in infection risk, but research on inequalities in the specific context of tracing apps during the pandemic is largely missing.

The aim of this study is therefore to analyze the sociodemographic differences in the use and in usability of the app using data from a German study on CWA. Results are likely to improve our understanding of barriers to download and user fidelity in different groups.

## Materials and methods

### Data collection procedure and participants

COVID-19 Snapshot Monitoring (COSMO) started on March 3, 2020. COSMO consists of consecutive cross-sectional surveys (waves) of the general population aged 18 to 74 years in Germany [17, 18]. Participants were recruited and paid by an ISO 26362:2009-compliant online panel (respondi.de) to match the distribution of the German population regarding age, gender, and residency in German federal states. Data was collected by online questionnaire. Data for each wave was collected within 38 hours (10am until 12pm the following day). Participants received a small monetary compensation for participation. As of January 23, 2021, 33 waves have been conducted. For this study, we used data from wave 15 to 19 conducted June 23, July 7, July 21, August 4, and August 18, since these the waves included questions regarding CWA. To put this time frame into perspective, 7-day incidence per 100.000 was 4.7 on June 23, 2.9 on July 7, 3.4 on July 21, 5.6 on August 4, and 8.9 on August 18, with an average percentage of positive test results of 0.82% (week 26 to 34, range 0.59%-1.02%) [19, 20]. During that time, nonpharmaceutical measures were generally eased except for contact tracing, mask mandate and regulations for quarantine, intercontinental travel and large gatherings. A total of 4960 participants were included (mean age 45.9 years, standard deviation 16.0, 50.4% female), 993 from wave 15, 1010 from wave 16, 1001 from wave 17, 999 from wave 18, and 957 from wave 19. As sociodemographic characteristics and CWA use did not vary substantially across waves, we report mainly results for data pooled across waves, except for usability questions which were only presented in single waves.

Ethical clearance was obtained from the University of Erfurt’s institutional review board (#20200302/20200501). Consent was suitably informed and obtained in written form. The study did not include minors under age 18.

### Measures

The complete questionnaires per wave are available from https://www.psycharchives.org/handle/20.500.12034/2397. The questionnaire is adapted bi-weekly by members of the consortium and contains continuously collected variables (e.g. risk perception) as well as items about the current pandemic situation. The questionnaire is pretested with about 30–50 participants before the panel is invited to participate.

CWA download among persons with a smartphone was assessed by the question “Have you already downloaded the app”. Response options were “Yes”, “No”, “The app is not compatible with my smartphone”. For multiple analyses, the category ‘not compatible’ was set to missing. As a sensitivity analysis, this group was also combined with the No group.

Age in years was first assessed as discrete numeric variable. Level of education was categorized into 0–9 years of schooling, at least 10 years of schooling without higher education entrance qualification, and higher education entrance qualification. Income was defined as net equivalized household income. Participants were also asked about any confirmed, unconfirmed, or past SARS-CoV-2 infection.

Additional questions on CWA included information on use, functionality, usability, and consequences of the app adapted from the system usability scale (SUS [21]). In the survey of June 23 (wave 15) items were administered to persons who did not download (e.g. “CWA is probably easy to use”, “[…] is probably compliant with data protection law.”, “[…] helps with infection containment.”, “[…] will be used by many people.”). Cronbach’s alpha shows sufficient reliability for the four items (α = .765). In the survey of August 18 (wave 19), participants who had confirmed download were given the items of the adapted SUS scale (“is easy to use”, “is easy to install”, “positive test result is easy to disclose”, “The app is doing a good job.”). Cronbach’s alpha claims for sufficient reliability (α = .731). Because additional items were collected that did not result from the SUS scale, we decided to present item based results instead of SUS-scale means (Table 3).

### Statistical analysis

We calculated means for continuous variables and percentages for categorical variables.

Main dichotomized outcome parameter for CWA use was the question “Have you already downloaded the app (yes/no)”. We originally decided only to analyze these two options, but further explored if and how regression estimates changed when the “not compatible” group was added either to the yes or no category.

Multiple logistic regression analyses were used to assess the association of potential predictors (sociodemographic characteristics, presence of chronic disease, work status, e.g., healthcare worker, wave) as independent variables on CWA use. Predictors were analyzed using logistic regression implementing recommendations by Royston and Sauerbrei [22] for model selection. Variables were chosen by backward selection (p<0.05 to stay) while simultaneously checking the functional form of the continuous covariate age, using the iterative multivariable fractional polynomial approach. Stepwise inclusion and exclusion of covariates is repeated, once the best functional form for the continuous covariates has been found. The resulting functional form of the variable age was a higher order polynomial. To increase interpretability and to increase comparability to other studies, we decided to categorize age into three brackets (18–39, 40–64, 65+). To investigate potential heterogeneousness of waves, wave was included as a dummy variable.

Usability of CWA was analyzed stratified by age, gender, and education. Significance was set on a test-wise 5% level.

We applied the SAS macro %mfp8 (http://mfp.imbi.uni-freiburg.de/software) for the multivariable fractional polynomial approach. SAS V9.4 (SAS Institute Inc., Cary, NC, USA) was used for all analyses.

## Results

Of all participants, 95.3% owned a smartphone and 36.5% had downloaded CWA (31.8% download in wave 15, 40.7% in wave 16, 38.0% in wave 17, 37.9% in wave 18, 33.9% in wave 19). Of all smartphone users in the study, 7.5% reported that CWA was not compatible with their device. This percentage did not vary much across waves. Persons who had downloaded CWA were significantly older than those who had not (mean age 46.2 years vs 43.8 years). A confirmed present, not yet confirmed or past infection was reported by 2.1% of participants. Additional information on sociodemographic variables is shown in Table 1.

**Table 1:** Participants’ sociodemographic characteristics of wave 15 to 19 of the COVID-19 Snapshot Monitoring (COSMO) surveys.

	Total		Corona Warn App (CWA) download							
			No smartphone		Yes		No		CWA not compatible	
	N	%	N	%	N	%	N	%	N	%
Total	4960	100.0	234	4.7	1810	36.5	2562	51.7	354	7.1
Wave	4960									
15 (June 23, 2020)	993	20.0	54	23.1	316	17.5	546	21.3	77	21.8
16 (July 7, 2020)	1010	20.4	42	17.9	411	22.7	477	18.6	80	22.6
17 (July 21, 2020)	1001	20.2	44	18.8	380	21.0	506	19.8	71	20.1
18 (August 4, 2020)	999	20.1	41	17.5	379	20.9	525	20.5	54	15.3
19 (August, 18, 2020)	957	19.3	53	22.6	324	17.9	508	19.8	72	20.3
Age (years)										
18 to 39	1976	39.8	32	13.7	706	39.0	1142	44.6	96	27.1
40 to 64	2185	44.1	109	46.6	784	43.3	1129	44.1	163	46.0
65+	799	16.1	93	39.7	320	17.7	291	11.4	95	26.8
Gender	4960									
Female	2501	50.4	102	43.6	860	47.5	1358	53.0	181	51.1
Male	2459	49.6	132	56.4	950	52.5	1204	47.0	173	48.9
Education	4960									
Up to 9 years of schooling	586	11.8	60	25.6	161	8.9	320	12.5	45	12.7
At least 10 years without higher entrance qualification	1638	33.0	83	35.5	531	29.3	916	35.8	108	30.5
At least 10 years with higher entrance qualification	2736	55.2	91	38.9	1118	61.8	1326	51.8	201	56.8
Net income of household	4514									
under 1250 Euro	634	12.8	56	23.9	161	8.9	362	14.1	55	15.5
1250 to under 1750 Euro	603	12.2	34	14.5	172	9.5	336	13.1	61	17.2
1750 to under 2250 Euro	664	13.4	25	10.7	214	11.8	382	14.9	43	12.1
2250 to under 3000 Euro	920	18.5	40	17.1	330	18.2	474	18.5	76	21.5
3000 to under 4000 Euro	843	17.0	27	11.5	347	19.2	421	16.4	48	13.6
4000 to under 5000 Euro	490	9.9	12	5.1	248	13.7	202	7.9	28	7.9
7000 Euro and more	360	7.3	8	3.4	200	11.0	137	5.3	15	4.2
Community size	4960									
Up to 5.000 inhabitants	801	16.1	44	18.8	247	13.6	457	17.8	53	15.0
5001 to 20.000 inhabitants	1129	22.8	49	20.9	399	22.0	596	23.3	85	24.0
20.001 to 100.000 inhabitants	1258	25.4	59	25.2	495	27.3	621	24.2	83	23.4
100.001 to 500.000 inhabitants	815	16.4	42	17.9	324	17.9	390	15.2	59	16.7
over 500.000 inhabitants	957	19.3	40	17.1	345	19.1	498	19.4	74	20.9
Household size	4952									
1 person (the respondent)	1322	26.7	100	42.7	463	25.6	644	25.1	115	32.5
2 persons	2041	41.1	103	44.0	760	42.0	1024	40.0	154	43.5
3–4 persons	1358	27.4	25	10.7	498	27.5	763	29.8	72	20.3
More than 4 persons	231	4.7	5	2.1	88	4.9	128	5.0	10	2.8
Lives in one of the five eastern federal states										
Yes	800	16.1	41	17.5	178	9.8	529	20.6	52	14.7
No	4160	83.9	193	82.5	1632	90.2	2033	79.4	302	85.3
Parents of respondent and respondent born in Germany	4936									
Yes	737	14.9	21	9.0	265	14.6	411	16.0	40	11.3
No	4199	84.7	208	88.9	1540	85.1	2140	83.5	311	87.9
Household language	4960									
Other than German	1069	21.6	50	21.4	362	20.0	596	23.3	61	17.2
German	3891	78.4	184	78.6	1448	80.0	1966	76.7	293	82.8
Chronic disease	4820									
present	1758	35.4	113	48.3	655	36.2	839	32.7	151	42.7
absent	3062	61.7	113	48.3	1116	61.7	1645	64.2	188	53.1
Respondent is health care professional	4960									
Yes	417	8.4	13	5.6	152	8.4	228	8.9	24	6.8
No	4543	91.6	221	94.4	1658	91.6	2334	91.1	330	93.2
Belonging to a minority group *	4788									
Yes	551	11.1	24	10.3	173	9.6	318	12.4	36	10.2
No	4237	85.4	198	84.6	1599	88.3	2129	83.1	311	87.9

*Minority group identity was self-reported by the question: “Do you perceive yourself to be part of a minority group within the country that you live in?”.

Participants were more likely to have downloaded CWA if they were male, 65 years and older, had at least 10 years of schooling with higher education entrance qualification, lived in a town or city with over 20,000 inhabitants, lived in one of the Western federal states of Germany (including the city of Berlin), or had a net household income of 4000 Euro and above. Persons who identified themselves as belonging to a minority group and persons whose main language was other than German were less likely to have downloaded the app. Adjusted odds ratios of are shown in Table 2. Please refer to S1 File for univariate estimates.

**Table 2:** Predictors of download of the Corona Warn App (n = 3762)*. Results of the multivariable logistic regression model. Odds Ratios below 1 indicate a decreased probability of download as compared to the reference group, odds ratios above 1 indicate increased probability.

Variable	Odds Ratio [95% confidence interval]
Age (reference 65+)	
18 to 39	0.473 [0.382;0.587]
40 to 64	0.566 [0.461;0.694]
Net household income < 4000 Euro (reference > = 4000)	0.514 [0.434;0.609]
Education (reference 10+ with higher entrance qualification)	
Up to 9 years of schooling	0.547 [0.433;0.691]
At least 10 years without higher entrance qualification	0.694 [0.595;0.809]
Belonging to a minority group (reference not belonging)	0.766 [0.616;0.954]
Household language other than German (reference no)	0.831 [0.704;0.979]
Community size up to 20,000 inhabitants (reference > 20,000)	0.857 [0.745;0.986]
Male gender (reference female)	1.162 [1.015;1.331]
Chronic disease present (reference absent)	1.235 [1.066;1.431]
Lives in one of the 10 western federal states or Berlin	2.313 [1.899;2.818]

* all participants with compatible smartphone.

Of those who had downloaded the app, 91.7% found that CWA was easy to install, 87.7% found CWA easy to use, and 61.4% thought that CWA is doing a good job (not downloaded: 13.4%). See Table 3 for detailed description.

**Table 3:** Information on use, functionality, and consequences of Corona Warn App (CWA). Items were selectively applied in single waves. Some items were administered either to persons who had confirmed download (“is easy to use”, “is easy to install”, “Positive test result is easy to disclose”) or who did not download (“is probably easy to use”).

	Total		CWA download							
			No smartphone		Yes		No		CWA not compatible	
	N	%	N	%	N	%	N	%	N	%
I would upload a positive test result	957	100.0	53	100.0	324	100.0	508	100.0	72	100.0
Do not agree	336	35.1	27	50.9	25	7.7	264	52.0	20	27.8
Agree/fully agree	621	64.9	26	49.1	299	92.3	244	48.0	52	72.2
CWA helps to protect me from infecting others	957	100.0	53	100.0	324	100.0	508	100.0	72	100.0
Do not agree	514	53.7	26	49.1	117	36.1	342	67.3	29	40.3
Agree/fully agree	443	46.3	27	50.9	207	63.9	166	32.7	43	59.7
CWA is easy to install	957	100.0	53	100.0	324	100.0	508	100.0	72	100.0
Do not agree	27	2.8	0	0	27	8.3	0	0	0	0
Agree/fully agree	297	31.0	0	0	297	91.7	0	0	0	0
CWA is easy to use (June 23)	993	100.0	54	100.0	316	100.0	546	100.0	77	100.0
Do not agree	29	2.9	0	0	29	9.2	0	0	0	0
Agree/fully agree	287	28.9	0	0	287	90.8	0	0	0	0
CWA is probably easy to use (June 23)	993	100.0	54	100.0	316	100.0	546	100.0	77	100.0
Do not agree	318	32.0	35	64.8	0	0	261	47.8	22	28.6
Agree/fully agree	359	36.2	19	35.2	0	0	285	52.2	55	71.4
CWA is easy to use (August 18)	957	100.0	53	100.0	324	100.0	508	100.0	72	100.0
Do not agree	40	4.2	0	0	40	12.3	0	0	0	0
Agree/fully agree	284	29.7	0	0	284	87.7	0	0	0	0
Positive test result is easy to disclose	957	100.0	53	100.0	324	100.0	508	100.0	72	100.0
Do not agree	112	11.7	0	0	112	34.6	0	0	0	0
Agree/fully agree	212	22.2	0	0	212	65.4	0	0	0	0
Persons important to me think I should use the app	957	100.0	53	100.0	324	100.0	508	100.0	72	100.0
Do not agree	659	68.9	41	77.4	124	38.3	439	86.4	55	76.4
Agree/fully agree	298	31.1	12	22.6	200	61.7	69	13.6	17	23.6
People using the app have a better image	957	100.0	53	100.0	324	100.0	508	100.0	72	100.0
Do not agree	835	87.3	47	88.7	252	77.8	474	93.3	62	86.1
Agree/fully agree	122	12.7	6	11.3	72	22.2	34	6.7	10	13.9
CWA is doing a good job	957	100.0	53	100.0	324	100.0	508	100.0	72	100.0
Do not agree	659	68.9	44	83.0	125	38.6	440	86.6	50	69.4
Agree/fully agree	298	31.1	9	17.0	199	61.4	68	13.4	22	30.6
I cannot explain the usefulness of the app	957	100.0	53	100.0	324	100.0	508	100.0	72	100.0
Do not agree	691	72.2	37	69.8	265	81.8	337	66.3	52	72.2
Agree/fully agree	266	27.8	16	30.2	59	18.2	171	33.7	20	27.8
CWA complies to data protection laws	993	100.0	54	100.0	316	100.0	546	100.0	77	100.0
Do not agree	491	49.4	35	64.8	53	16.8	373	68.3	30	39.0
Agree/fully agree	502	50.6	19	35.2	263	83.2	173	31.7	47	61.1

Of participants who had downloaded the app, 96.2% (wave 16 and 17) confirmed that they would report a positive test result by upload into the app (not downloaded: 52.0%). This percentage decreased in wave 19 (92.3%, not downloaded: 48.0%). Willingness to disclose was significantly higher in older adults and increased with higher educational degrees and income (see also Table 4 in S1 File).

Participants of wave 17 (n = 1001) responded to the question “Would you quarantine for 14 days if the app gave you the information of a high-risk contact?”. Of those who had downloaded the app, 60.3% indicated that they would quarantine after receiving the information from the app; of those who had not downloaded the app, 35.5% would definitely quarantine. Willingness to quarantine increased significantly with age (63.3% of those aged 32 and younger, 83.9% of participants older than 60).

Eighty three percent of participants with download expressed their trust that CWA complied with data protection laws (not downloaded: 31.7%). Trust in data protection compliance was again significantly higher in older adults and in adults with higher income (see also Table 3 in S1 File).

Descriptive statistics and sensitivity analyses showed that the group whose smartphone was not compatible was very similar to the group that had downloaded the app (see Tables 1 and 3). Adding the “not compatible” group to the response option “no” or “yes” changed regression estimates slightly but had no substantial differential effect on results. Also, including survey wave as a covariate had no substantial effect on results (see S1 File).

## Discussion

Success of mobile phone tracing apps for containment in pandemic emergencies depends both on a sufficiently high number of downloads and active users as well as on an equal access of all societal groups [7]. This survey based on an online panel studied the reach of the national tracing app in Germany and found that in total 37% of the adult study population had downloaded the German Corona Warn App (CWA) between June and August 2020. Higher education, income, and age independently increased the likelihood for download, increased trust in data protection, and increased the willingness to cooperate, namely, to disclose a positive test result to the app, and to self-quarantine.

The percentage of downloads found in this study is in line with findings for other national tracing apps in countries where installation regime was not mandatory, 37% for the Australian COVIDSafe app launched in April 2020, [23] 38% for the first version of the British NHS contact tracing app on the Isle of Wight [24], 40% for the Rakning C-19 app in Iceland, [25] and 44% for a representative sample of the Swiss population [26]. Our findings are also in line with 36% found by a representative telephone survey of a sample of 1018 persons aged 14 and older that was conducted in November 2020 in Germany (Kantar Sample, [27]). Download statistics of CWA indicated an increase from 18 Mio end of August 2020 to 23.5 Mio on Dec 3, 2020, and to 25.4 Mio on Feb 5, 2021 in Germany [6].

In our survey, persons who had downloaded CWA were significantly older than those who had not, as opposed to other studies [27]. This difference may partly be explained by our older, more digitally affine sample. Yet, a recent study evaluating CWA use in Germany also found that older persons were more likely to download the app [28]. An age gradient towards a higher percentage of downloads in older age groups was also found for the initial phase of app deployment in Australia [23] which suggests vulnerability as motivation. The idea of vulnerability also aligns with our finding that persons with chronic disease were significantly more likely to download CWA, independently of age. Enthusiasm for the app may also be triggered by the misunderstanding that the app can detect if infected persons are in the proximity [23].

Higher education and income were major indicators for download in our study, independently of technical preconditions. This finding closely matches indicators for the SwissCovid app [26], and results from other studies in Germany [27, 28]. This inequality is particularly worrying as CWA could have the highest public health benefits when used by those with high infection risk, i.e. persons who have to work and live in close quarters, and use public transport [13, 14]. Inequality in downloads might partly due to one initial access barrier, namely that CWA was only installable on mobile phone with the newest operating system and was only available in German. For CWA this issue has subsequently been recognized and resolved by increased compatibility with older systems and a multi-language interface.

Our data also shows a divide between West and East (with lower use in the former GDR federal states as compared to the western states), rural and urban areas, and language. Arguably, the underlying factor may be health literacy, or the lack thereof, but a number of additional factors need to be considered. Among these, control aversion, i.e. the mistrust in governmental actions because of past experience under the former coercive regime of East Germany, has been mentioned [29]. Likewise, an analysis of the early phase of the pandemic in the US showed that conspiracy beliefs were more frequent in younger adults with low social and educational status, and conspiracy beliefs were strong indicators for insufficient protective behavior such as mask wearing [30]. An earlier German study found that trust in others was indeed indicating CWA use [31]. Additionally, trust in the government was a major predictor in a multi-country survey investigating the theoretical willingness to install a tracing app [32].

Our study also shed some light on the perception of consequences of use of CWA, namely that positive tests need to be uploaded and that the notification of an epidemiologically relevant contact may indicate the need to quarantine, a certain risk for severe disease and death. In our study, over 92% of persons with download reported that they would disclose a positive test result, as compared to just 48% of persons without download. In reality, 59% of app users with positive test results had uploaded their result between September and February 2021 [6]. Fear of consequences has indeed been mentioned as a reason to reject contact tracing apps [32]. Persons at working age and persons in precarious working situations might not be able to afford voluntary quarantine due to an exposure notification and might therefore be less inclined to download the app.

Lower health literacy may again be one of the reasons that participants who did not download the app, expressed apparent mistrust in data protection. This finding is especially remarkable because, after having supported a privacy-preserving central data storage solution which had caused considerable indignation in public, Germany had adopted the decentralized approach. Here, data is stored parsimoniously and uniquely on the user’s mobile device, not on any central server. In contrast to apps deployed e.g. in China, South-Korea and India, CWA does not store geolocation data. Lack of data privacy and the feeling of being watched was also one of the most frequently mentioned reasons not to use CWA in the Kantar Sample [27]. It comes to mind that concerns about data protection issues might also have been put forward as an easy and socially acceptable reason not to use a tracing app. Recent research suggests that app design choices (e.g. perceived security and privacy risks, location use) might not be as relevant as compared to sociodemographic status of potential users, their readiness for technology, and their perception of public health benefits [33]. Regarding public health benefits, unsurprisingly, our data confirm that a considerable part of non-users were not aware of usefulness and effectiveness of CWA. This points at missed communication opportunities.

One main limitation of our study is that we relied on self-reported data of an online panel. It is unknown whether participants really kept the app on their mobile phone or if they actively opened, updated, and used the app when installed. Also, there is a tendency towards higher education and older mean age in the COSMO samples compared to census data. Still, our results align well with results from surveys from other countries and other German representative surveys, and to estimates from German health authorities. The timing of our survey from June to August 2020 is another limitation. Nevertheless, our results regarding sociodemographic characteristics of CWA users were confirmed by a subsequent representative German survey from November 2020. Research questions about fidelity and effectiveness of CWA could be addressed more directly if a follow-up of confirmed app users were possible, e.g. to investigate prospectively the proportion of positive test results among app users who had received a self-isolation recommendation from the app [24]. However, the timing of our investigation can also be seen as an advantage, as it allowed us to study genuine preventive behaviour in a low-risk situation.

This study supports the hypothesis of a digital divide that separates users and non-users of CWA along a well-known health gap of education, income, urbanity and region. Principles of equity must therefore guide not only communication about CWA but also its implementation and deployment strategies.

## Supporting information

S1 File Tables with supporting information. (PDF)
