# Supplementary material for: Sociodemographic characteristics determine download and use of a Corona contact tracing app in Germany—Results of the COSMO surveys
Source: PLoS One. 2021 Sep 2;16(9):e0256660. doi: 10.1371/journal.pone.0256660 (PMC8412249; doi:10.1371/journal.pone.0256660)
Supplement: S1 File — (PDF) [file pone.0256660.s001.pdf]

S1 File. Tables with supporting information

Table 1. Results of logistic regressions for the **univariate** association of variables with download of the Corona Warn App. Odds Ratios below 1 indicate a decreased probability of download as compared to the reference group, odds ratios above 1 indicate increased probability.

Variable	Odds Ratio [95% confidence interval]
Age (reference 65+)	
18 to 39	0.562 [0.468;0.676]
40 to 64	0.631 [0.526;0.758]
Net household income < 4000 Euro (reference >=4000)	0.469 [0.400;0.549]
Education (reference 10+ with higher entrance qualification)	
Up to 9 years of schooling	0.597 [0.486;0.733]
At least 10 years without higher entrance qualification	0.688 [0.602;0.786]
Belonging to a minority group (reference not belonging)	0.724 [0.595;0.881]
Household language other than German (reference no)	0.825 [0.712;0.955]
Community size up to 20,000 inhabitants (reference > 20,000)	0.795 [0.702;0.901]
Male gender (reference female)	1.246 [1.104;1.406]
Chronic disease present (reference absent)	1.151 [1.013;1.307]
Lives in one of the 10 western federal states or Berlin	2.386 [1.989;2.862]
Wave (reference wave 19)	
15	0.907 [0.746;1.104]
16	1.351 [1.115;1.637]
17	1.177 [0.971;1.428]
18	1.132 [0.934;1.371]

Table 2. Results of logistic regression for the **multivariable** association of variables with download of the Corona Warn App. Wave has been included in the final models. Odds Ratios below 1 indicate a decreased probability of download as compared to the reference group, odds ratios above 1 indicate increased probability.

Variable	Odds Ratio [95% confidence interval]
Age (reference 65+)	
18 to 39	0.473 [0.381;0.587]
40 to 64	0.563 [0.459;0.691]
Net household income < 4000 Euro (reference >=4000)	0.513 [0.433;0.608]
Education (reference 10+ with higher entrance qualification)	
Up to 9 years of schooling	0.549 [0.434;0.695]
At least 10 years without higher entrance qualification	0.691 [0.592;0.806]
Belonging to a minority group (reference not belonging)	0.755 [0.606;0.941]
Household language other than German (reference no)	0.837 [0.71;0.988]
Community size up to 20,000 inhabitants (reference > 20,000)	0.858 [0.746;0.988]
Male gender (reference female)	1.159 [1.012;1.328]
Chronic disease present (reference absent)	1.227 [1.058;1.422]
Lives in one of the 10 western federal states or Berlin	2.327 [1.909;2.836]
Wave (reference wave 19)	
15	0.939 [0.756;1.166]
16	1.417 [1.145;1.753]
17	1.231 [0.994;1.526]
18	1.105 [0.893;1.367]

Table 3. Predictors of trust in data protection compliance of the Corona Warn App (n=868, wave 15); Odds Ratios > 1 indicate predictors of trust.

Variable	Odds Ratio [95% confidence interval]
Age (reference 65+)	
18 to 39	0.544 [0.355;0.833]
40 to 64	0.611 [0.407;0.916]
Net household income < 4000 Euro (reference >=4000)	0.624 [0.434;0.896]
Education (reference 10+ with higher entrance qualification)	
Up to 9 years of schooling	0.687 [0.434;1.087]
At least 10 years without higher entrance qualification	0.776 [0.571;1.055]
Belonging to a minority group (reference not belonging)	0.826 [0.504;1.352]
Household language other than German (reference no)	0.750 [0.543;1.036]
Community size up to 20,000 inhabitants (reference > 20,000)	1.138 [0.854;1.515]
Male gender (reference female)	1.105 [0.840;1.453]
Chronic disease present (reference absent)	0.868 [0.643;1.172]
Lives in one of the 10 western federal states or Berlin	1.477 [1.019;2.139]

Table 4. Predictors of willingness to disclose a positive test result via Corona Warn App (n=1511, waves 16 and 17).Odds Ratios > 1 indicate predictors of willingness to disclose.

Variable	Odds Ratio [95% confidence interval]
Age (reference 65+)	
18 to 39	0.500 [0.336;0.744]
40 to 64	0.618 [0.422;0.905]
Net household income < 4000 Euro (reference >=4000)	0.577 [0.411;0.810]
Education (reference 10+ with higher entrance qualification)	
Up to 9 years of schooling	0.395 [0.268;0.582]
At least 10 years without higher entrance qualification	0.482 [0.370;0.628]
Belonging to a minority group (reference not belonging)	0.684 [0.484;0.966]
Household language other than German (reference no)	0.727 [0.548;0.964]
Community size up to 20,000 inhabitants (reference > 20,000)	0.961 [0.752;1.229]
Male gender (reference female)	1.242 [0.978;1.576]
Chronic disease present (reference absent)	1.103 [0.855;1.422]
Lives in one of the 10 western federal states or Berlin	1.472 [1.092;1.985]
Wave (16 vs 17)	1.079 [0.852;1.366]

Table 5. Estimated regression coefficients, their standard error, p-values, and confidence intervals, measures of goodness of fit for the logistic model corresponding to Table 2

Analysis of Maximum Likelihood Estimates

Parameter		DF	Estimate	Standard Error	Wald Chi-Square	Pr > ChiSq
Intercept		1	-0.5514	0.0842	42.8669	<.0001
Age (reference 65+)	18 to 39	1	-0.3096	0.0530	34.1696	<.0001
	40 to 64	1	-0.1335	0.0491	7.3981	0.0065
Net household income < 4000 Euro (reference >=4000)		1	-0.3330	0.0432	59.4034	<.0001
Education (reference 10+ with higher entrance qualification)	Up to 9 years of schooling	1	-0.2839	0.0765	13.7601	0.0002
	At least 10 years without higher entrance qualification	1	-0.0403	0.0560	0.5186	0.4715
Belonging to a minority group (reference not belonging)		1	-0.1308	0.0557	5.5107	0.0189
Household language other than German (reference no)		1	-0.0938	0.0420	4.9906	0.0255
Community size up to 20,000 inhabitants (reference > 20,000)		1	-0.0755	0.0357	4.4614	0.0347
Male gender (reference female)		1	0.0737	0.0345	4.5633	0.0327
Chronic disease present (reference absent)		1	0.1051	0.0375	7.8352	0.0051
Lives in one of the 10 western federal states or Berlin		1	0.4209	0.0503	69.9300	<.0001

Model Fit Statistics

Criterion	Intercept Only	Intercept and Covariates
AIC	5157.462	4912.351
SC	5163.698	4987.185
-2 Log L	5155.462	4888.351

Table 6. Estimated regression coefficients, their standard error, p-values, and confidence intervals, measures of goodness of fit for the logistic model corresponding to Table 2

Analysis of Maximum Likelihood Estimates						
**Parameter**		**DF**	**Estimate**	**Standard Error**	**Wald Chi-Square**	**Pr > ChiSq**
Intercept		1	-0.5581	0.0846	43.5450	<.0001
Age (reference 65+)	18 to 39	1	-0.3083	0.0531	33.6601	<.0001
	40 to 64	1	-0.1360	0.0492	7.6288	0.0057
Net household income < 4000 Euro (reference >=4000)		1	-0.3342	0.0433	59.5272	<.0001
Education (reference 10+ with higher entrance qualification)	Up to 9 years of schooling	1	-0.2791	0.0768	13.2171	0.0003
	At least 10 years without higher entrance qualification	1	-0.0449	0.0562	0.6377	0.4245
Belonging to a minority group (reference not belonging)		1	-0.1383	0.0560	6.1043	0.0135
Household language other than German (reference no)		1	-0.0899	0.0421	4.5511	0.0329
Community size up to 20,000 inhabitants (reference > 20,000)		1	-0.0748	0.0359	4.3515	0.0370
Male gender (reference female)		1	0.0724	0.0346	4.3734	0.0365
Chronic disease present (reference absent)		1	0.1014	0.0377	7.2556	0.0071
Lives in one of the 10 western federal states or Berlin		1	0.4238	0.0504	70.5952	<.0001
Wave (reference wave 19)	15	1	-0.1792	0.0692	6.7063	0.0096
	16	1	0.2293	0.0677	11.4793	0.0007
	17	1	0.0904	0.0683	1.7495	0.1859
	18	1	-0.0184	0.0676	0.0737	0.7860

Model Fit Statistics		
**Criterion**	**Intercept Only**	**Intercept and Covariates**
**AIC**	5157.462	4901.802
**SC**	5163.698	5001.581
**-2 Log L**	5155.462	4869.802

Table 7. Estimated regression coefficients, their standard error, p-values, and confidence intervals, measures of goodness of fit for the logistic model corresponding to Table 3 Trust

Analysis of Maximum Likelihood Estimates						
**Parameter**		**DF**	**Estimate**	**Standard Error**	**Wald Chi-Square**	**Pr > ChiSq**
Intercept		1	-0.0309	0.1706	0.0328	0.8563
Age (reference 65+)	18 to 39	1	-0.2418	0.1061	5.1937	0.0227
	40 to 64	1	-0.1257	0.0984	1.6324	0.2014
Net household income < 4000 Euro (reference >=4000)		1	-0.2359	0.0924	6.5208	0.0107
Education (reference 10+ with higher entrance qualification)	Up to 9 years of schooling	1	-0.1660	0.1483	1.2527	0.2630
	At least 10 years without higher entrance qualification	1	-0.0437	0.1090	0.1606	0.6886
Belonging to a minority group (reference not belonging)		1	-0.0957	0.1258	0.5786	0.4469
Household language other than German (reference no)		1	-0.1440	0.0824	3.0534	0.0806
Community size up to 20,000 inhabitants (reference > 20,000)		1	0.0645	0.0731	0.7793	0.3774
Male gender (reference female)		1	0.0499	0.0698	0.5105	0.4749
Chronic disease present (reference absent)		1	-0.0707	0.0766	0.8512	0.3562
Lives in one of the 10 western federal states or Berlin		1	0.1948	0.0946	4.2443	0.0394

Model Fit Statistics		
**Criterion**	**Intercept Only**	**Intercept and Covariates**
**AIC**	1203.270	1195.050
**SC**	1208.037	1252.244
**-2 Log L**	1201.270	1171.050

Table 8. Estimated regression coefficients, their standard error, p-values, and confidence intervals, measures of goodness of fit for the logistic model corresponding to Table 4 Disclosure

Analysis of Maximum Likelihood Estimates						
**Parameter**		**DF**	**Estimate**	**Standard Error**	**Wald Chi-Square**	**Pr > ChiSq**
Intercept		1	0.7808	0.1417	30.3616	<.0001
Age (reference 65+)	18 to 39	1	-0.3024	0.0950	10.1421	0.0014
	40 to 64	1	-0.0890	0.0890	1.0016	0.3169
Net household income < 4000 Euro (reference >=4000)		1	-0.2749	0.0865	10.0934	0.0015
Education (reference 10+ with higher entrance qualification)	Up to 9 years of schooling	1	-0.3765	0.1232	9.3343	0.0022
	At least 10 years without higher entrance qualification	1	-0.1764	0.0901	3.8319	0.0503
Belonging to a minority group (reference not belonging)		1	-0.1900	0.0882	4.6357	0.0313
Household language other than German (reference no)		1	-0.1594	0.0720	4.8931	0.0270
Community size up to 20,000 inhabitants (reference > 20,000)		1	-0.0198	0.0626	0.1003	0.7515
Male gender (reference female)		1	0.1082	0.0608	3.1645	0.0753
Chronic disease present (reference absent)		1	0.0488	0.0650	0.5646	0.4524
Lives in one of the 10 western federal states or Berlin		1	0.1934	0.0763	6.4252	0.0113
Wave	16 vs. 17	1	0.0380	0.0602	0.3982	0.5280

Model Fit Statistics		
**Criterion**	**Intercept Only**	**Intercept and Covariates**
**AIC**	1760.676	1696.030
**SC**	1765.997	1765.197
**-2 Log L**	1758.676	1670.030
